# Outcomes of Trachelectomy vs. Hysterectomy for Early-Stage Cervical Cancer: A Systematic Review and Meta-Analysis

**DOI:** 10.3389/fsurg.2021.735944

**Published:** 2021-11-11

**Authors:** Juan Guo, Qingwei Hu, Zaixing Deng, Xiaotian Jin

**Affiliations:** ^1^Department of Obstetrics and Gynecology, Huzhou Maternity & Child Health Care Hospital, Huzhou, China; ^2^Department of Pathology, Huzhou Maternity & Child Health Care Hospital, Huzhou, China

**Keywords:** radical hysterectomy, radical trachelectomy, abdominal trachelectomy, vaginal trachelectomy, early stage cervical cancer, meta-analysis

## Abstract

**Objective:** To provide updated evidence on comparative efficacy for clinical outcomes of radical trachelectomy and radical hysterectomy in patients with early-stage cervical cancer.

**Methods:** A systematic search was conducted in the PubMed, Scopus, Cochrane Database of Systematic Reviews, and Google scholar databases. Studies were done in patients with early-stage cervical cancer that compared the outcomes between radical trachelectomy (RT) and hysterectomy (RH) were considered for inclusion in the review. The outcomes of interest were operative time, the volume of blood loss, need for blood transfusion, any complications, length of hospital stay, risk of recurrence, and survival. The strength of association was presented in the form of pooled relative risk (RR), hazards risk (HR), and weighted mean difference (WMD). Statistical analysis was done using STATA version 16.0.

**Results:** A total of 12 articles were included in the meta-analysis. The majority were retrospective cohort-based studies. Compared to RH, the operative time (in min) was comparatively higher in RT (WMD 23.43, 95% CI: 5.63, 41.24). Patients undergoing RT had blood loss (in ml) similar to those undergoing RT (WMD −81.34, 95% CI: −170.36, 7.68). There were no significant differences in the risk of intra-operative (RR 1.61, 95% CI: 0.49, 5.28) and post-operative complications (RR 1.13, 95% CI: 0.54, 2.40) between the two groups. Patients in the RT group had lesser duration of post-operative hospital stay (in days) (WMD −1.65, 95% CI: −3.22, −0.09). There was no statistically significant difference in the risk of recurrence (HR 1.21, 95% CI: 0.68, 2.18), 5-year overall survival (HR 1.00, 95% CI: 0.99, 1.02), and recurrence-free survival (HR 0.99, 95% CI: 0.96, 1.01) between the two groups.

**Conclusion:** Among the patients with early-stage cervical cancer, RT is similar to RH in safety and clinical outcomes. Future studies with a randomized design and larger sample sizes are needed to further substantiate these findings.

## Introduction

One of the most common causes of mortality in young women aged 20–39 years is cervical cancer (1, 2). In 2018, cervical cancer was ranked as fourth most frequently diagnosed cancer and was attributed to an estimated 0.5 million cases and ~3,00,000 deaths worldwide (3). With the widespread implementation of cervical cancer screening programs, the proportion of people diagnosed with early-stage cancer is increasing. Until recently, the preferred treatment for such patients has been radical hysterectomy along with lymphadenectomy (4). The downside to this management modality is that fertility is compromised (4).

Radical trachelectomy (RT) consists of removing most of the portion of the cervix and the upper part of the vagina (5). As the uterus is left intact, there is a possibility of preserving fertility. This is usually done for those with early-stage cancer, that is, those with Stage IA1, stage IA2, and stage IB1 and in those with lesions <2 cm and with a negative lymph node metastasis status (6). The approaches to perform RT could be vaginal or abdominal, and the procedure is usually accompanied by pelvic node dissection (6, 7). In a recent systematic review that analyzed 1,300 RTs, there were nearly 300 pregnancies with 173 live births reported (8). The review found a 3% risk of cancer recurrence and the most reported complications were miscarriage and chorioamnionitis (8). The review concluded that RT could be a safe option for women with early cervical cancer to preserve their fertility.

While there are studies comparing the clinical outcomes between RH and RT, very few attempts have been done to systematically synthesize the findings of these studies to derive a meaningful interpretation of the comparative efficacy of these two modalities. Prodromidou et al. compared outcomes of abdominal RT and RH by pooling data from five studies consisting of 840 women with early-stage cervical cancer (9). The authors excluded data of all vaginal trachelectomies. They noted a significantly prolonged operative time for RT compared to RH, but the 5-year overall survival and disease-free survival were similar between the two groups. Based on these findings, the authors concluded that RT has a similar efficacy and safety profile compared to RH and recommended the use of this technique for women of reproductive age who wish to have future pregnancies (9). Another recent review, using five studies, concluded that RT, particularly the vaginal mode, is safe and effective for the management of early-stage cervical cancer with lesion size of <2 cm and negative tumor margins (10). However, with new studies being published, there is a need to update the evidence and therefore, the current meta-analysis was undertaken.

## Materials and Methods

### Search Strategy

A thorough systematic search of English language articles published until April 30, 2021, was carried out in electronic search engines, such as PubMed, Scopus, Cochrane Database of Systematic Reviews, and Google academic databases. Supplementary Table 1 has the specific details of the search strategy used to identify the relevant literature for this meta-analysis. The literature search aimed at identifying studies done in patients with early-stage cervical cancer that compared the outcomes between trachelectomy and hysterectomy. The outcomes of interest were operative time, volume of blood loss, need for blood transfusion, any complications, length of hospital stay, risk of recurrence, and survival. The study processes were in compliance with the Preferred Reporting Items for Systematic Reviews and Meta-analyses (PRISMA) guidelines.

### Selection Criteria and Methods

The search strategy was executed in the databases mentioned. Studies identified through these databases were compared and duplicates were removed. Subject experts (QH, ZD) from the study team screened the titles and abstracts as an initial step. After removing the articles that were considered not useful for inclusion in the review, the full texts of the remaining articles were reviewed in detail. In case of any disagreements between the two study authors with respect to the inclusion or exclusion of studies, a third senior experienced author was consulted, and consensus was reached through discussions. Only studies that fulfilled the inclusion criteria were included in the meta-analysis. In order to identify additional literature, the reference list of the included studies was also reviewed.

### Inclusion Criteria

Studies that were randomized controlled trials or adopted a cohort approach or retrospective data-based studies were considered for inclusion. For a study to be included, it should have been done in patients with early-stage cervical cancer and had compared the outcomes between trachelectomy and hysterectomy. The outcomes of interest were operative time, volume of blood loss, need for blood transfusion, any complications, length of hospital stay, risk of recurrence, and survival.

### Exclusion Criteria

Review articles were excluded. Studies that did not provide data on the outcomes of interest or did not compare the outcomes between hysterectomy and trachelectomy were excluded.

### Data Extraction and Quality Assessment

Through the use of a pretested data extraction sheet, two study authors separately extracted data from the included studies. The methodological assessment was done independently by the two authors using the Newcastle–Ottawa Quality Assessment Scale (11).

### Statistical Analysis

This meta-analysis, using STATA version 16.0 (StataCorp), reported effect sizes as pooled relative risk (RR) or hazards ratio (HR) with 95% CI for categorical outcomes and weighted mean difference (WMD) for continuous outcomes. *I*^2^ was used to assess the heterogeneity and in instances where the value of *I*^2^ exceeded 40%, the random effects model was used (12). Sub-group analysis was done based on the route of trachelectomy, that is, vaginal or abdominal. For reporting statistical significance, a *p* < 0.05 was considered. Egger's test was employed to assess the presence or absence of publication bias for categorical outcomes (13).

## Results

### Selection of Articles, Study Characteristics, and Quality of Included Studies

**Figure 1:**
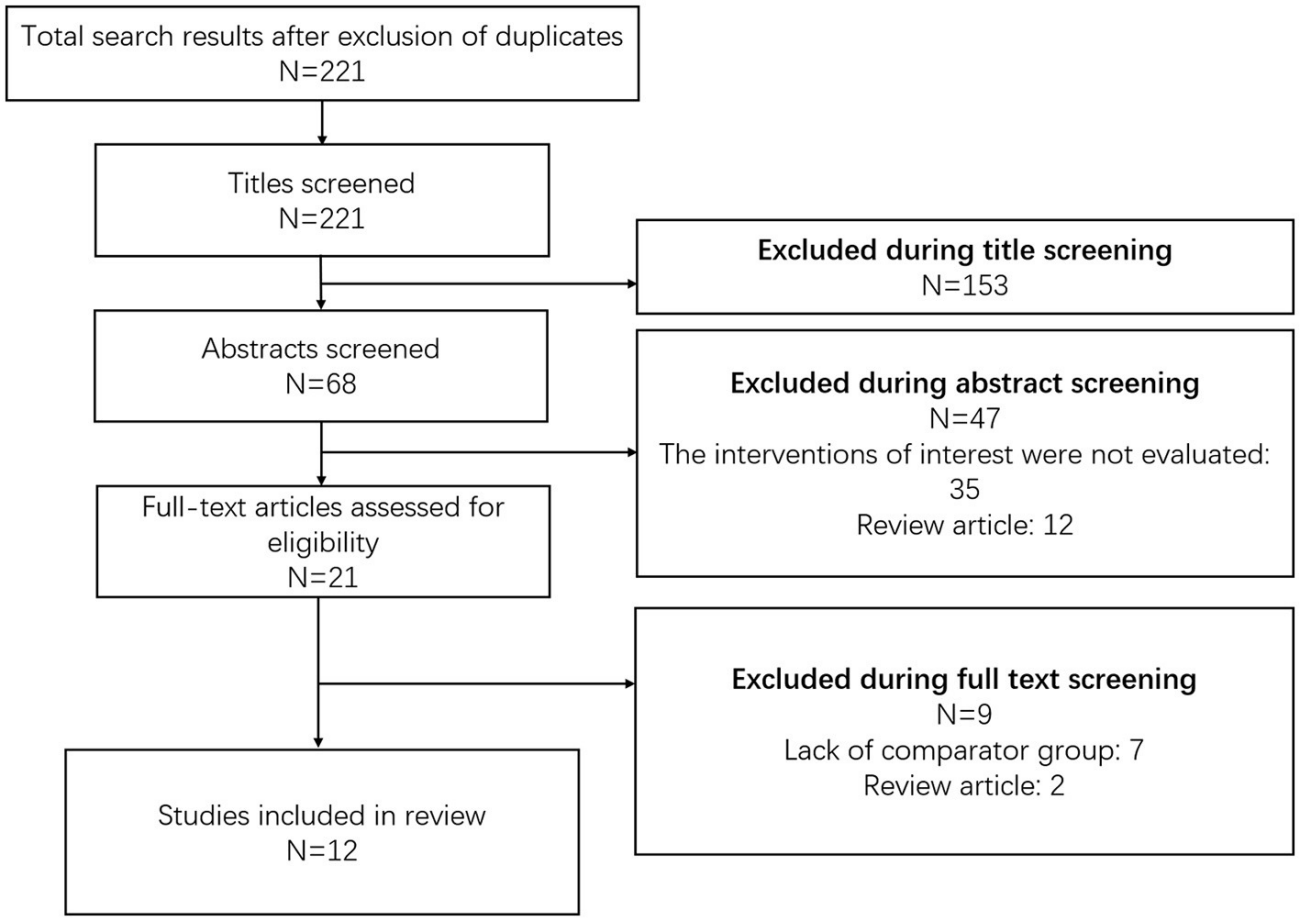
The selection process of the studies included in the review.

Using the search strategy and after removal of the duplicates, a total of 221 citations were retrieved (Figure 1). Screening of the titles led to the removal of 153 studies. Of the remaining 68 citations, 47 were omitted after reading the abstract. The remaining 21 articles were reviewed in detail and finally, 12 articles were included in the meta-analysis (14–25). Supplementary Table 3 presents the details of the included studies. All the studies were non-randomized and majority were retrospective cohort-based studies (*n* = 6/12) followed by case-control studies (*n* = 3/12). Most of the studies were done in China (*n* = 4/12) followed by the United States (*n* = 2/12) and Japan (*n* = 2/12). One study each was conducted in Canada, France, the United Kingdom, and The Netherlands. Vaginal trachelectomy was reported in four studies and abdominal trachelectomy was reported in the remaining eight studies. A detailed description of the surgical procedures adopted for radical trachelectomy or radical hysterectomy in the included studies has been presented in Supplementary Table 2. A need for adjuvant treatment, either chemotherapy or radiation therapy or a combination of both, was reported in less than one-third of the participants in most of the included studies (Supplementary Table 2). Lymph node surgical assessment was done in all the studies. Open surgical approach to radical hysterectomy was used in most of the studies. Most of the studies had a mean follow-up period of at least 36 months. The results of the quality evaluation of the included studies are provided in Supplementary Table 2. The included studies were of good quality.

### Operative Time and Blood Loss

**Figure 2:**
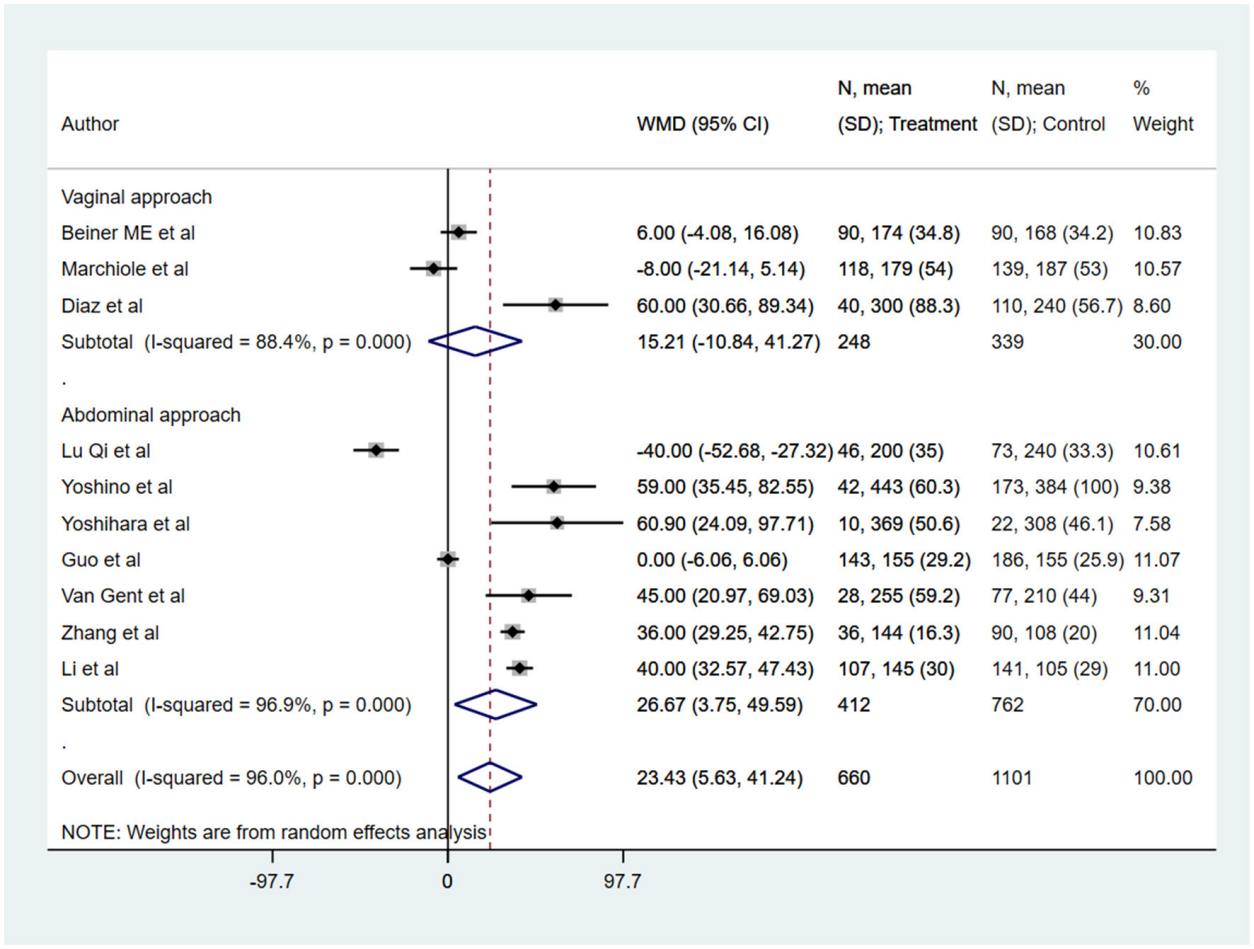
The pooled effect size for the operative time (in min) is based on comparison between radical trachelectomy and hysterectomy in patients with early-stage cervical cancer.

Compared to radical hysterectomy (RH), the operative time (in min) was comparatively higher in radical trachelectomy (RT) (WMD 23.43, 95% CI: 5.63, 41.24; *I*^2^ = 96.0%, *N* = 10) (Figure 2). In the subgroup analysis, the operative time was similar for trachelectomy and hysterectomy when the approach for trachelectomy was vaginal (WMD 15.21, 95% CI: −10.84, 41.27; *I*^2^ = 88.4%, *N* = 3). However, with the abdominal approach for trachelectomy, the operative time was higher compared to hysterectomy (WMD 26.67, 95% CI: 3.75, 49.59; *I*^2^ = 96.9%, *N* = 7) (Figure 2).

**Figure 3:**
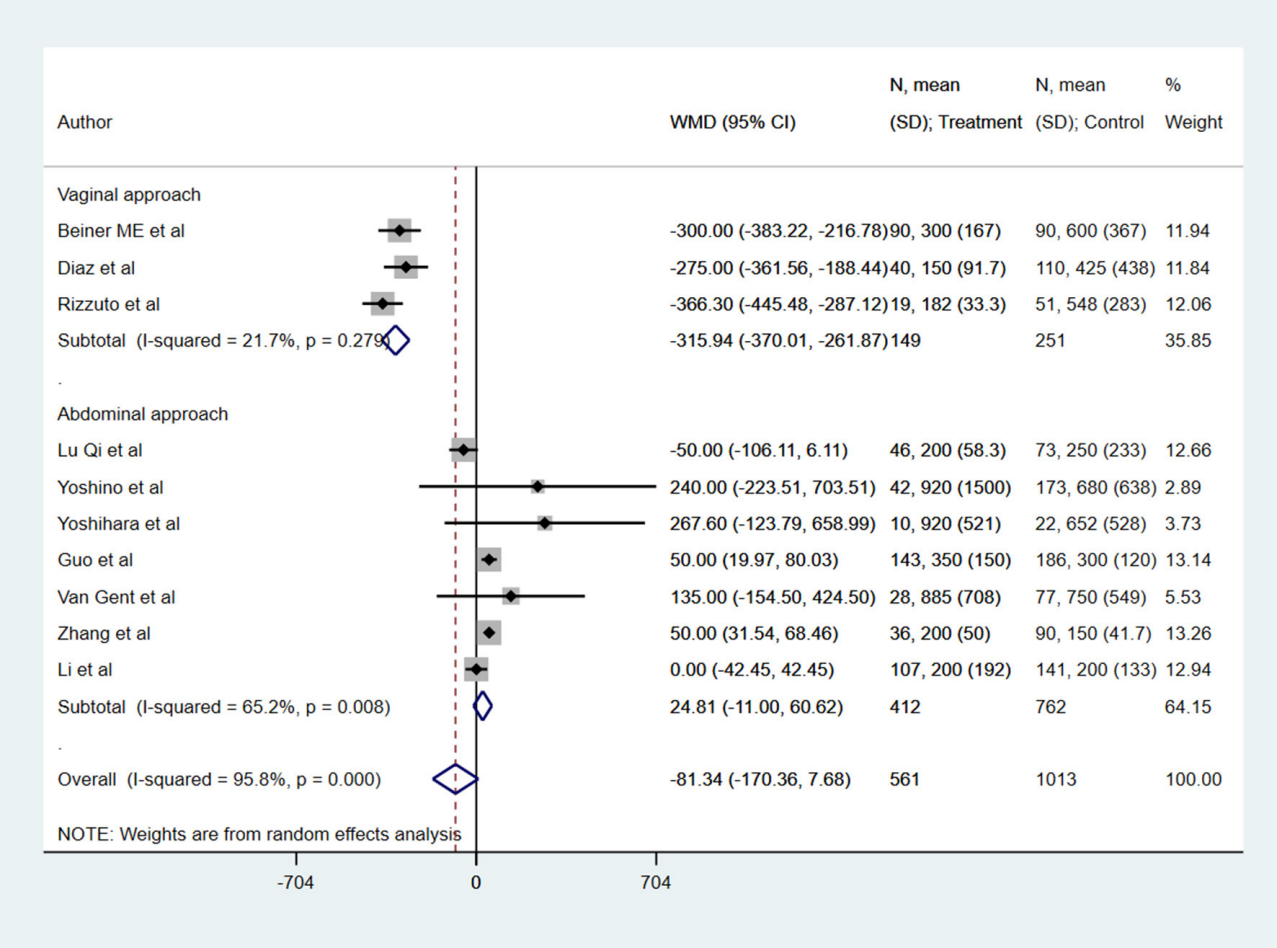
The pooled effect size for the blood loss (in ml) is based on the comparison between radical trachelectomy and hysterectomy in patients with early-stage cervical cancer.

With respect to blood loss (in ml), patients undergoing radical trachelectomy had blood loss similar to those undergoing radical hysterectomy (WMD −81.34, 95% CI: −170.36, 7.68; *I*^2^ = 95.8%, *N* = 10) (Figure 3). In the subgroup analysis, vaginal trachelectomy was associated with comparatively lesser blood loss (WMD −315.94, 95% CI: −370.01, −261.87; *I*^2^ = 21.7%, *N* = 3), whereas no significant difference was noted between radical abdominal trachelectomy and hysterectomy (WMD 24.81, 95% CI: −11.0, 60.62; *I*^2^ = 65.2%, *N* = 7) (Figure 3).

### Complication, Need for Blood Transfusion, and Length of Hospital Stay

**Figure 4:**
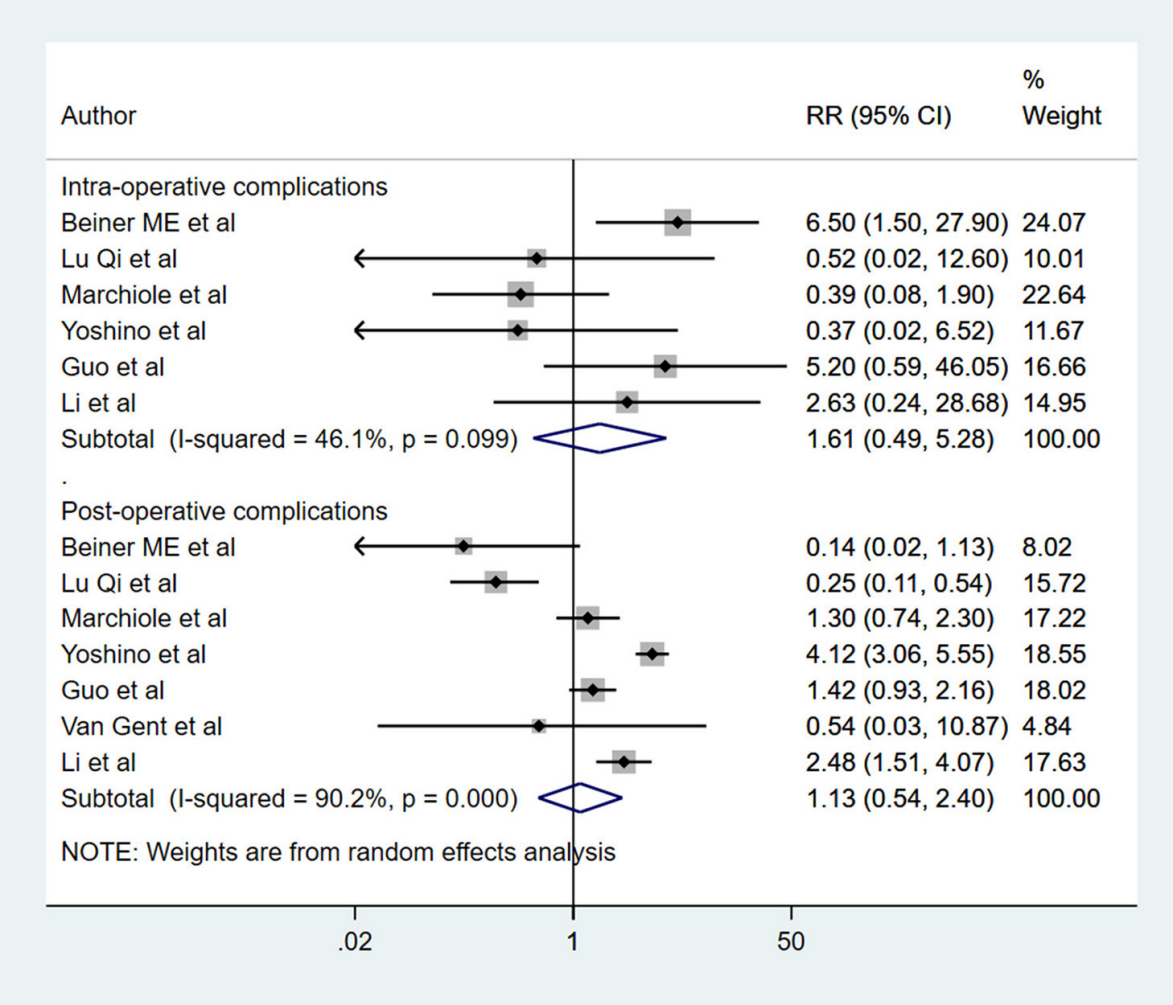
The pooled effect size for the intra-operative and post-operative complications based on the comparison between radical trachelectomy and hysterectomy in patients with early-stage cervical cancer.

**Figure 5:**
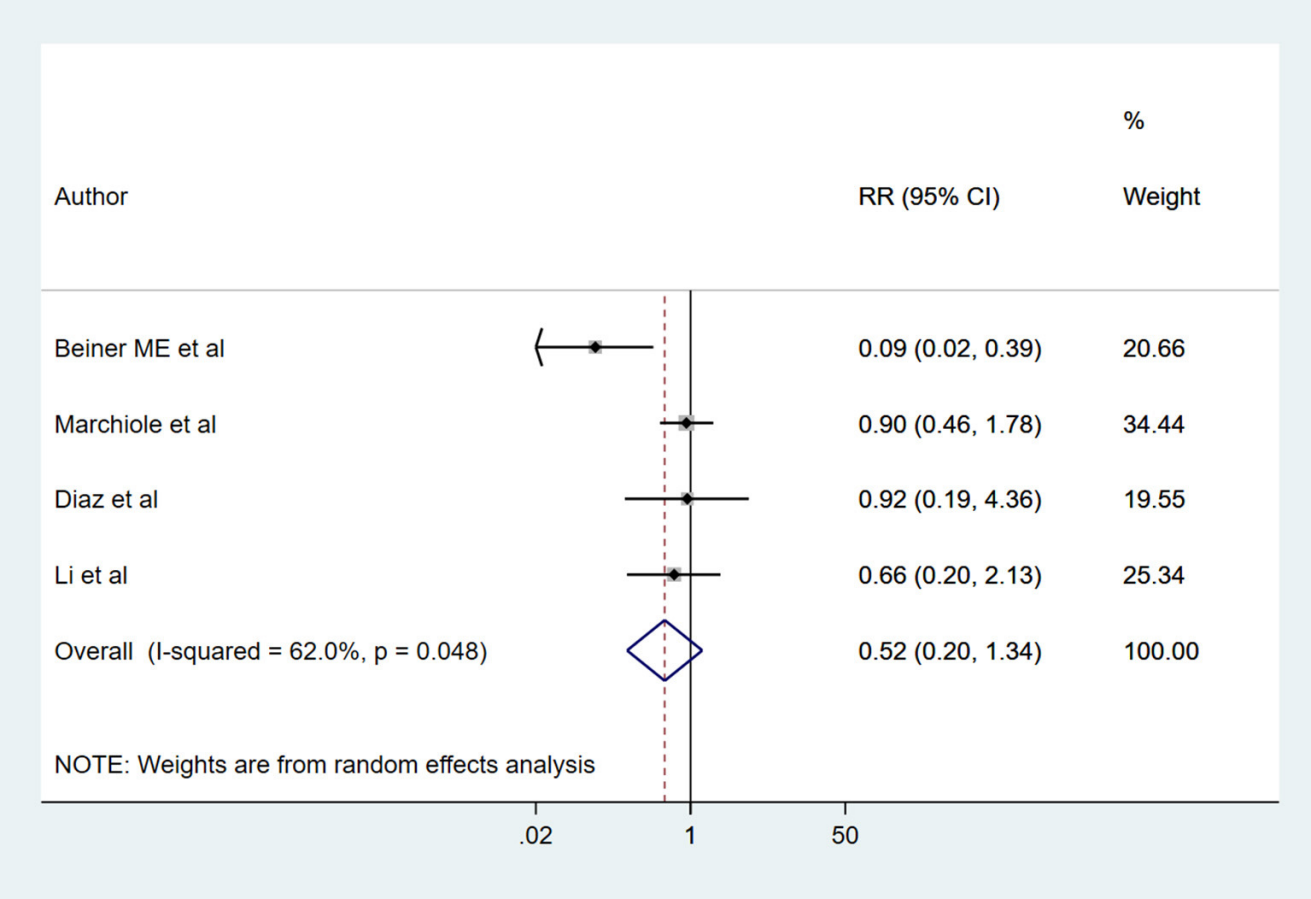
The pooled effect size for the need for blood transfusion based on the comparison between radical trachelectomy and hysterectomy in patients with early-stage cervical cancer.

**Figure 6:**
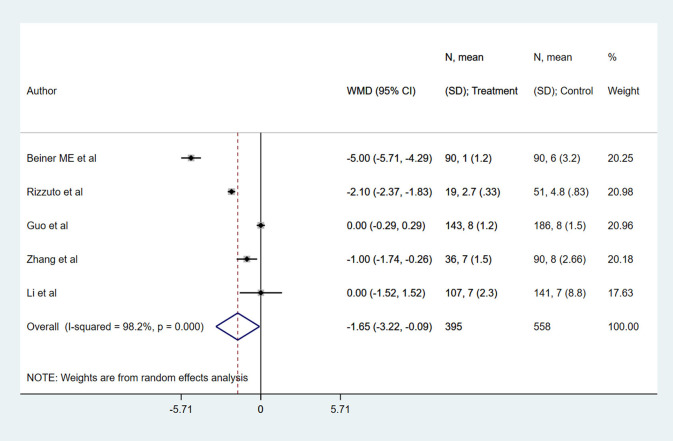
The pooled effect size for the length of post-operative hospital stay (in days) based on the comparison between radical trachelectomy and hysterectomy in patients with early-stage cervical cancer.

There were no significant differences in the risk of intra-operative (RR 1.61, 95% CI: 0.49, 5.28; *I*^2^ = 46.1%, *N* = 6) and post-operative complications (RR 1.13, 95% CI: 0.54, 2.40; *I*^2^ = 90.2%, *N* = 7) between RT and RH groups (Figure 4). The risk of the need for blood transfusion was similar for both RT and RH groups (RR 0.52, 95% CI: 0.20, 1.34; *I*^2^ = 62.0%, *N* = 4) (Figure 5). However, those with RT had lesser duration of post-operative hospital stay (in days) (WMD −1.65, 95% CI: −3.22, −0.09; *I*^2^ = 98.2%, *N* = 5) (Figure 6). Egger's test did not indicate the presence of publication bias (*P* = 0.17 for risk of complications; *P* = 0.91 for risk of the need for blood transfusion). In the subgroup analysis, there were no differences in the risk of complications and need for blood transfusion based on vaginal or abdominal trachelectomy (Table 1). However, in those with vaginal trachelectomy, the length of post-operative hospital stay was significantly lesser than RH (WMD −3.53, 95% CI: −6.37, −0.69; *I*^2^ = 98.2%, *N* = 2), but no such difference was noted for those with abdominal trachelectomy (WMD −0.35, 95% CI: −1.09, 0.40; *I*^2^ = 67.5%, *N* = 3) (Table 1).

**Table 1:** Subgroup analysis based on the approach of trachelectomy (vaginal and abdominal).

	**Pooled effect size with 95% confidence interval**	
	**Vaginal approach**	**Abdominal approach**
Intra-operative complications	*N =* 2; *I*^2^ = 84.7% RR 1.62 (95% CI: 0.10, 25.5)	*N =* 4; *I*^2^ = 0.0% RR 1.74 (95% CI: 0.48, 6.34)
Post-operative complications	*N =* 2; *I*^2^ = 77.0% RR 0.53 (95% CI: 0.06, 4.52)	*N =* 5; *I*^2^ = 92.1% RR 1.37 (95% CI:0.56, 3.34)
Need for blood transfusion	*N =* 3; *I*^2^ = 74.6% RR 0.45 (95% CI:0.11, 1.84)	*N =* 1 RR 0.66 (95% CI:0.20, 2.13)
Recurrence	*N =* 3; *I*^2^ = 7.2% HR 2.99 (95% CI:1.24, 7.18)^*^	*N =* 4; *I*^2^ = 0.0% HR 0.59 (95% CI:0.27, 1.30)
5-year survival rate	*N =* 3; *I*^2^ = 0.0% HR 0.98 (95% CI:0.96, 1.01)	*N =* 6; *I*^2^ = 0.0% HR 1.01 (95% CI:1.00, 1.03)
5-year recurrence free survival rate	*N =* 3; *I*^2^ = 32.0% HR 0.98 (95% CI:0.95, 1.01)	*N =* 5; *I*^2^ = 0.0% HR 0.99 (95% CI:0.96, 1.02)
Post-operative stay	*N =* 2; *I*^2^ = 98.2% WMD−3.53 (95% CI: −6.37, −0.69)^*^	*N =* 3; *I*^2^ = 67.5% WMD−0.35 (95% CI: −1.09, 0.40)

* *Significant at P < 0.05*.

### Recurrence, 5-Year Overall Survival, and 5 Years Recurrence-Free Survival

**Figure 7:**
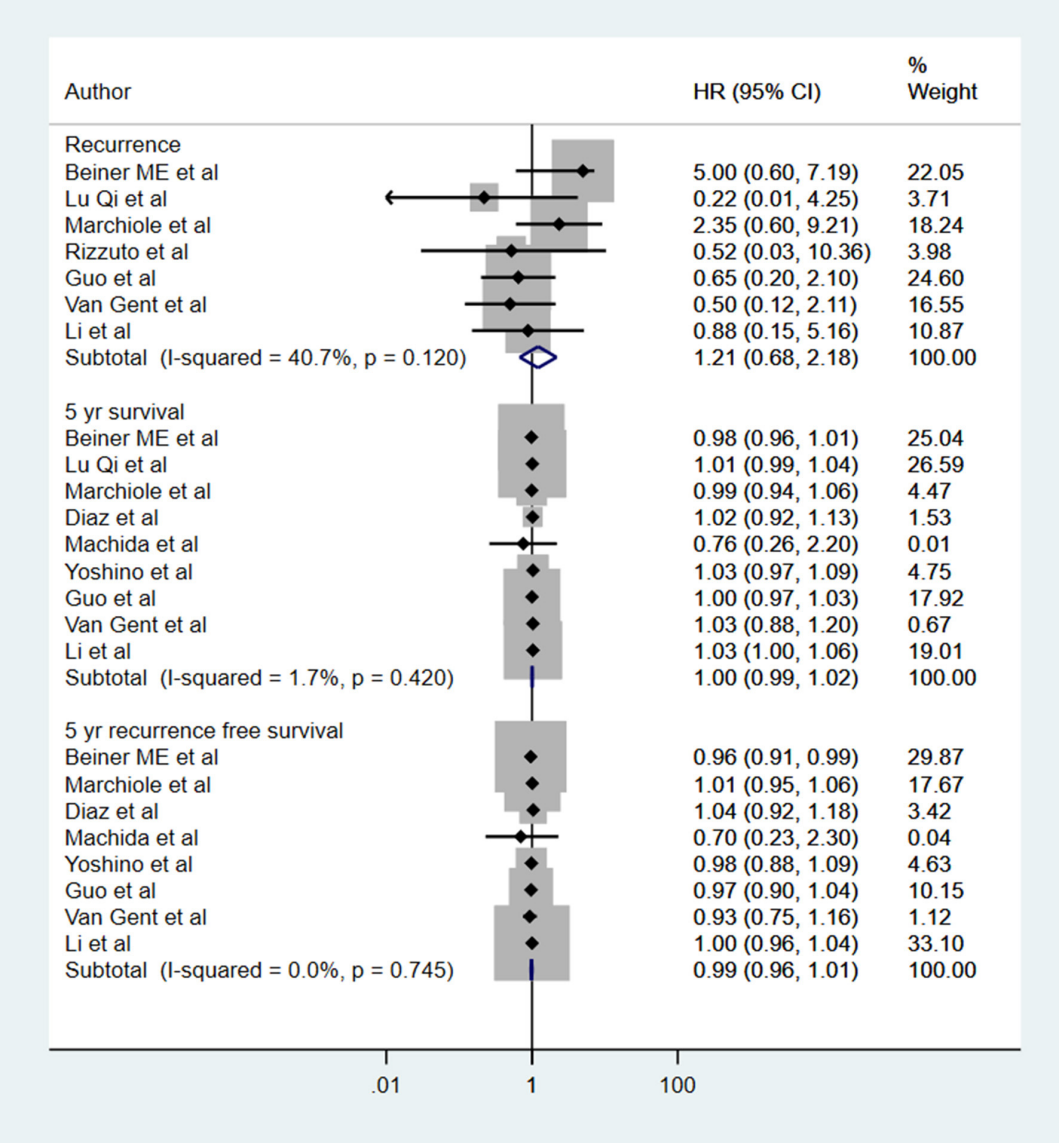
The pooled effect size for the recurrence, 5-year overall survival, and 5 years recurrence-free survival based on comparison between radical trachelectomy and hysterectomy in patients with early-stage cervical cancer.

There was no statistically significant difference in the risk of recurrence among the two groups of patients (HR 1.21, 95% CI: 0.68, 2.18; *I*^2^ = 40.7%, *N* = 7). The 5-year overall survival (HR 1.00, 95% CI: 0.99, 1.02; *I*^2^ = 1.7%, *N* = 9) and recurrence-free survival (HR 0.99, 95% CI: 0.96, 1.01; *I*^2^ = 0.0%, *N* = 8) was similar in the two groups (Figure 7). Egger's test did not indicate the presence of publication bias (*P* = 0.35 for recurrence; *P* = 0.19 for overall survival and *P* = 0.93 for recurrence-free survival). In the subgroup analysis, the recurrence rate was higher in those with vaginal (HR 2.99, 95% CI: 1.24, 7.18; *I*^2^ = 7.2%, *N* = 3) but not in those with abdominal trachelectomy (HR 0.59, 95% CI: 0.27, 1.30; *I*^2^ = 0.0%, *N* = 4) (Table 1). The 5-year overall survival and recurrence-free survival were similar for both vaginal and abdominal trachelectomy compared to radical hysterectomy.

## Discussion

The current meta-analysis was conducted to provide updated evidence on the safety and efficacy of radical trachelectomy, compared to radical hysterectomy in patients with early-stage cervical cancer. The available studies suggest that the feasibility and success of conservative trachelectomy surgery depends largely on the selection of the patients, personal history (nulliparous or not), size of the tumor, as well as the status of lymph node localization at the preoperative stage (26, 27). Our review found an increased operative time in patients undergoing RT compared to patients undergoing RH. The blood loss, rates of complications, recurrence rate, 5-year overall survival, and recurrence-free survival were similar among the two groups. RT patients had a lower duration of post-operative hospital stay. These results are similar to the previous reviews on this aspect and add to the body of the already available evidence (9, 10). Our findings underscore that in women of reproductive age with early-stage cervical cancer, RT can be used in place of RH as a way to preserve fertility.

The most optimal approach to RT, that is, vaginal or abdominal, is still under research and is influenced by the preferences and the skills of the gynecological surgeon. In our sub-group analysis based on the approach of RT, the operative time was higher with the abdominal approach. Vaginal approach was associated with lesser blood loss and lesser duration of post-operative hospital stay, but the rate of recurrence was also higher compared to RH. These findings are somewhat similar to a case-control study by Cao et al. (28) that compared outcomes between vaginal and abdominal RT using a total of 150 women with early cervical cancer and reported similar outcomes in both groups. However, the rate of recurrence was higher in vaginal RT (10%) compared to abdominal RT (no recurrence) and the pregnancy rate was significantly higher in vaginal RT (around 40%) compared to abdominal RT (around 9.0%) (28).

One of the reasons for the higher rate of recurrence in vaginal trachelectomy, compared to the abdominal route, might be that performing trachelectomy through the vaginal route requires the treating surgeon to have immense expertise and skills in performing laparoscopic lymphadenectomy and vaginal radical surgery (28). On the other hand, the skills, training, and instruments required to conduct abdominal trachelectomy are more or less similar to abdominal radical hysterectomy, and therefore, many of the trained gynecological oncology surgeons could perform this procedure with ease (29, 30). Furthermore, studies have shown comparable rates of recurrence between vaginal and abdominal RT in tumors with size ≤2 cm (28, 31). However, in tumors >2 cm, the recurrence rates are higher for the vaginal RT. In the study by Cao et al., no recurrence was noted in the abdominal RT groups, whereas the recurrence rate was higher in vaginal RT group (28). The authors noted that the sites of recurrence were mainly located in the parametrium tissue. The findings strongly suggests that one of the main reasons for reduced recurrence in cases of abdominal RT, particularly in tumors with size more than 2 cm, could be the feasibility of being able to access and remove wider parametrium (32).

A meta-analysis by Xu et al. included three clinical trials (*n* = 587) and showed no statistically significant difference between the RT and RH in recurrence rate, 5-year recurrence-free survival rate, 5-year overall survival rate, intra- or post-operative complications, and a need for blood transfusion (33). Additionally, RT was associated with reduced blood loss and shorter post-operative hospital stays. While these studies provide an initial indication of merits and demerits of each of these approaches, further randomized controlled trials with adequate sample size and robust methodology are needed. In almost all the included studies in the current meta-analysis, there was a mix of patients with varied tumor histology. In the majority of the studies, squamous cell carcinoma (SCC) was the most common histologic type. HPV load is a possible prognostic marker of high-grade squamous intraepithelial lesion and tropism of HPV for squamous epithelial cells of the exocervix may be a factor in favor of a conservative treatment with trachelectomy. Studies have shown that the need for chemoradiation is higher in cases of radical abdominal trachelectomy, as patients requiring this management modality also have adverse prognostic factors (10, 34, 35). While chemoradiation could improve prognosis, it could also adversely affect the reproductive organs and fertility.

One set of outcomes that we did not consider as part of this meta-analysis was pregnancy-related outcomes. Prior evidence has documented the overall pregnancy rate of around 16% after RT, as well as a pregnancy loss rate of ~20% and preterm labor rate of ~35% (36, 37). In a review by Willows et al. that included 1,238 women with early-stage cervical cancer that underwent RT (38), a total of 469 (37.8%) pregnancies were reported and ~65% of them resulted in live births (38). These findings underscore the point that in women with early-stage cervical cancer, who are still in their reproductive age group and want to preserve their fertility, RT could be a possible option. While RT has been shown, to some extent, to be effective and safe in preserving the fertility in women with small (under 2 cm in size) tumors, its efficiency in patients with large tumors (size between 2 and 4 cm) is relatively less studied. Few studies, such as those of Lintner et al. have shown favorable outcomes, particularly for overall survival, in patients with early-stage cervical cancer and with tumor size larger than 2 cm (39). An important issue to note is that most of these patients with large tumor sizes generally require adjuvant chemotherapy or radiotherapy and therefore will impact fertility negatively.

There are some limitations to this study. Most of the included studies were observational in nature, and therefore some degree of bias is expected. Furthermore, it is unclear whether the studies reported the effect sizes after adjustment for all the potential confounders. For some of the included studies, the baseline comparability of the two groups was not established, and therefore, the final outcomes could have been influenced by these baseline differences. We observed a modest degree of heterogeneity for some of the outcomes. Therefore, we conducted subgroup analysis by the approach for trachelectomy, that is, vaginal or abdominal to minimize some of the heterogeneity observed. Additionally, although the studies referred to early-stage cervical cancer, most of them included a heterogenous group of patients, that is, those with tumor size below 2 cm as well as between 2 and 4 cm. Also, the studied population was heterogenous in terms of tumor histology. The results were not separated based on tumor size and different tumor histotypes. It is well-known that the efficacy of radical trachelectomy is best with tumor size ≤ 2 cm in largest diameter. Large tumors have an unfavorable prognosis with respect to overall lymph nodal metastasis, patient survival, and recurrence rate (40, 41). This heterogeneity in patient tumor characteristics may make the results difficult to interpret. Additionally, larger tumors may often require adjuvant treatment, which may negatively affect fertility. Not all the studies provided information on the presence of micro- and macro-metastases and this prevents proper interpretation of the pooled findings. As the studies included were not randomized in design, there is a possibility that radical hysterectomy (the current standard treatment modality) was offered to patients with associated adverse prognostic factors, thereby rendering the findings of the comparisons biased. Recent evidence indicates that vaginal cuff closure reduces exposure to the peritoneum and consequently reduces the risk of recurrence (42, 43). Only a few studies in the included meta-analysis provided information about vaginal cuff during the surgery. Future studies should explore the role of the vaginal cuff in early-stage cervical cancer. Another critical limitation of the review is that the included studies did not report on the fertility/pregnancy outcomes, particularly the rate of pregnancies carried to term, and therefore, this aspect was not addressed in this review. Patients undergoing trachelectomy may have an increased number of complications during pregnancy, such as cervical incompetence, implantation difficulties, miscarriages, or premature births. Further studies and reviews are needed to compare the possibility of having a full-term pregnancy between the two treatment modalities.

## Conclusions

The current meta-analysis of observational studies suggests that radical trachelectomy could be similar to hysterectomy in terms of clinical outcomes, such as blood loss, rates of complications, recurrence rate, 5-year overall survival, and recurrence-free survival. Radical trachelectomy should, therefore, be considered in women of reproductive age who wish to preserve their fertility. Future studies and reviews are needed to focus on the chances of full-term pregnancy in patients undergoing radical trachelectomy. There is a need for large randomized controlled trials to further validate the findings of this review.

## Data Availability Statement

The raw data supporting the conclusions of this article will be made available by the authors, without undue reservation.

## Author Contributions

JG and QH conceived and designed the study and wrote the paper. ZD and XJ were involved in literature search, data collection, and reviewed and edited the manuscript. JG and ZD analyzed the data. All authors read and approved the final manuscript.

## Conflict of Interest

The authors declare that the research was conducted in the absence of any commercial or financial relationships that could be construed as a potential conflict of interest.

## Publisher's Note

All claims expressed in this article are solely those of the authors and do not necessarily represent those of their affiliated organizations, or those of the publisher, the editors and the reviewers. Any product that may be evaluated in this article, or claim that may be made by its manufacturer, is not guaranteed or endorsed by the publisher.

## References

[1] Small W Bacon MA Bajaj A Chuang LT Fisher BJ Harkenrider MM . Cervical cancer: a global health crisis. Cancer. (2017) 123:2404–12. 10.1002/cncr.30667 28464289

[2] Zhang S Xu H Zhang L Qiao Y. Cervical cancer: epidemiology, risk factors and screening. Chin J Cancer Res. (2020) 32:720–8. 10.21147/j.issn.1000-9604.2020.06.05 33446995 PMC7797226

[3] Bray F Ferlay J Soerjomataram I Siegel RL Torre LA Jemal A. Global cancer statistics 2018: GLOBOCAN estimates of incidence and mortality worldwide for 36 cancers in 185 countries. CA Cancer J Clin. (2018) 68:394–424. 10.3322/caac.21492 30207593

[4] Somashekhar SP Ashwin KR. Management of early stage cervical cancer. Rev Recent Clin Trials. (2015) 10:302–8. 10.2174/1574887110666150923113629 26411950

[5] Dargent D Mathevet P. Schauta's vaginal hysterectomy combined with laparoscopic lymphadenectomy. Baillieres Clin Obstet Gynaecol. (1995) 9:691–705. 10.1016/S0950-3552(05)80392-X 8821248

[6] Mejia-Gomez J Feigenberg T Feigenber T Arbel-Alon S Kogan L Benshushan A. Radical trachelectomy: a fertility-sparing option for early invasive cervical cancer. Isr Med Assoc J. (2012) 14:324–8. 22799068

[7] Ramirez PT Schmeler KM Soliman PT Frumovitz M. Fertility preservation in patients with early cervical cancer: radical trachelectomy. Gynecol Oncol. (2008) 110:S25–8. 10.1016/j.ygyno.2008.03.025 18501409

[8] Gizzo S Ancona E Saccardi C Patrelli TS Berretta R Anis O . Radical trachelectomy: the first step of fertility preservation in young women with cervical cancer (Review). Oncol Rep. (2013) 30:2545–54. 10.3892/or.2013.2736 24065029 PMC3839990

[9] Prodromidou A Iavazzo C Fotiou A Psomiadou V Douligeris A Vorgias G . Short- and long term outcomes after abdominal radical trachelectomy versus radical hysterectomy for early stage cervical cancer: a systematic review of the literature and meta-analysis. Arch Gynecol Obstet. (2019) 300:25–31. 10.1007/s00404-019-05176-y 31062151

[10] Feng Y Zhang Z Lou T Wang S Bai H Zhang Z. The security of radical trachelectomy in the treatment of IA-IIA cervical carcinoma requires further evaluation: updated meta-analysis and trial sequential analysis. Arch Gynecol Obstet. (2019) 299:1525–36. 10.1007/s00404-019-05141-9 31011877

[11] Wells G Shea B O'Connell D Peterson J Welch V Losos M . The Newcastle-Ottawa (NOS) for Assessing the Quality of Nonrandomized Studies in Meta-Analysis. Ottawa, ON: Ottawa Hospital Research Institute. Available online at: http://www.ohri.ca/programs/clinical_epidemiology/oxford.asp (accessed May 20, 2021).

[12] Higgins J Green S. Cochrane Handbook for Systematic Reviews of Interventions. Chichester, UK: John Wiley & Sons Ltd (2009).

[13] Egger M Davey Smith G Schneider M Minder C. Bias in meta-analysis detected by a simple, graphical test. BMJ. (1997) 315:629–34. 10.1136/bmj.315.7109.629 9310563 PMC2127453

[14] Beiner ME Hauspy J Rosen B Murphy J Laframboise S Nofech-Mozes S . Radical vaginal trachelectomy vs. radical hysterectomy for small early stage cervical cancer: a matched case-control study. Gynecol Oncol. (2008) 110:168–71. 10.1016/j.ygyno.2008.04.027 18539313

[15] Lu Q Zhang Z Xiao M Liu C Zhang Z. The surgical morbidity and oncological outcome of total laparoscopic radical trachelectomy versus total laparoscopic radical hysterectomy for early stage cervical cancer: a retrospective study with 11-year follow-up. Onco Targets Ther. (2019) 12:7941–7. 10.2147/OTT.S224525 31576149 PMC6769159

[16] Marchiole P Benchaib M Buenerd A Lazlo E Dargent D Mathevet P. Oncological safety of laparoscopic-assisted vaginal radical trachelectomy (LARVT or Dargent's operation): a comparative study with laparoscopic-assisted vaginal radical hysterectomy (LARVH). Gynecol Oncol. (2007) 106:132–41. 10.1016/j.ygyno.2007.03.009 17493666

[17] Diaz JP Sonoda Y Leitao MM Zivanovic O Brown CL Chi DS . Oncologic outcome of fertility-sparing radical trachelectomy versus radical hysterectomy for stage IB1 cervical carcinoma. Gynecol Oncol. (2008) 111:255–60. 10.1016/j.ygyno.2008.07.014 18755500

[18] Machida H Mandelbaum RS Mikami M Enomoto T Sonoda Y Grubbs BH . Characteristics and outcomes of reproductive-aged women with early-stage cervical cancer: trachelectomy vs hysterectomy. Am J Obstet Gynecol. (2018) 219:461.e1–8. 10.1016/j.ajog.2018.08.020 30138618 PMC6648708

[19] Rizzuto I MacNab W Nicholson R Nalam M Rufford B. Less radical surgery for women with early stage cervical cancer: our experience on radical vaginal trachelectomy and laparoscopic pelvic lymphadenectomy. Gynecol Oncol Rep. (2019) 28:65–7. 10.1016/j.gore.2019.03.005 30911594 PMC6416726

[20] Yoshino AI Kobayashi E Kodama M Hashimoto K Ueda Y Sawada K . Oncological and reproductive outcomes of abdominal radical trachelectomy. Anticancer Res. (2020) 40:5939–47. 10.21873/anticanres.14615 32988926

[21] Yoshihara K Ishiguro T Chihara M Shima E Adachi S Isobe M . The safety and effectiveness of abdominal radical trachelectomy for early-stage cervical cancer during pregnancy. Int J Gynecol Cancer. (2018) 28:782–7. 10.1097/IGC.0000000000001218 29498982 PMC5929493

[22] Guo J Zhang Y Chen X Sun L Chen K Sheng X. Surgical and oncologic outcomes of radical abdominal trachelectomy versus hysterectomy for stage IA2-IB1 cervical cancer. J Minim Invasive Gynecol. (2019) 26:484–91. 10.1016/j.jmig.2018.06.006 29908338

[23] van Gent MDJM van den Haak LW Gaarenstroom KN Peters AAW van Poelgeest MIE Trimbos JBMZ . Nerve-sparing radical abdominal trachelectomy versus nerve-sparing radical hysterectomy in early-stage (FIGO IA2-IB) cervical cancer: a comparative study on feasibility and outcome. Int J Gynecol Cancer. (2014) 24:735–43. 10.1097/IGC.0000000000000114 24651626

[24] Zhang D Li J Ge H Ju X Chen X Tang J . Surgical and pathological outcomes of abdominal radical trachelectomy versus hysterectomy for early-stage cervical cancer. Int J Gynecol Cancer. (2014) 24:1312–8. 10.1097/IGC.0000000000000185 24987922

[25] Li X Li J Wen H Ju X Chen X Xia L . The survival rate and surgical morbidity of abdominal radical trachelectomy versus abdominal radical hysterectomy for stage IB1 cervical cancer. Ann Surg Oncol. (2016) 23:2953–8. 10.1245/s10434-016-5216-1 27044448

[26] Rema P Ahmed I. Conservative surgery for early cervical cancer. Indian J Surg Oncol. (2016) 7:336–40. 10.1007/s13193-015-0476-y 27651696 PMC5016325

[27] Plante M Renaud MC François H Roy M. Vaginal radical trachelectomy: an oncologically safe fertility-preserving surgery. an updated series of 72 cases and review of the literature. Gynecol Oncol. (2004) 94:614–23. 10.1016/j.ygyno.2004.05.032 15350349

[28] Cao DY Yang JX Wu XH Chen YL Li L Liu KJ . Comparisons of vaginal and abdominal radical trachelectomy for early-stage cervical cancer: preliminary results of a multi-center research in China. Br J Cancer. (2013) 109:2778–82. 10.1038/bjc.2013.656 24169350 PMC3844910

[29] Hurst SA Del Priore G Ungar L Smith JR. Experiences in abdominal radical trachelectomy. Am J Obstet Gynecol. (2010) 202:e8–9. 10.1016/j.ajog.2009.11.006 20042173

[30] Sato S Aoki D Kobayashi H Saito T Nishimura R Nagano T . Questionnaire survey of the current status of radical trachelectomy in Japan. Int J Clin Oncol. (2011) 16:141–4. 10.1007/s10147-010-0146-6 21086011

[31] Nishio H Fujii T Kameyama K Susumu N Nakamura M Iwata T . Abdominal radical trachelectomy as a fertility-sparing procedure in women with early-stage cervical cancer in a series of 61 women. Gynecol Oncol. (2009) 115:51–5. 10.1016/j.ygyno.2009.06.036 19646742

[32] Einstein MH Park KJ Sonoda Y Carter J Chi DS Barakat RR . Radical vaginal versus abdominal trachelectomy for stage IB1 cervical cancer: a comparison of surgical and pathologic outcomes. Gynecol Oncol. (2009) 112:73–7. 10.1016/j.ygyno.2008.09.007 18973933 PMC4994890

[33] Xu L Sun FQ Wang ZH. Radical trachelectomy versus radical hysterectomy for the treatment of early cervical cancer: a systematic review. Acta Obstet Gynecol Scand. (2011) 90:1200–9. 10.1111/j.1600-0412.2011.01231.x 21718255

[34] Li J Wu X Li X Ju X. Abdominal radical trachelectomy: is it safe for IB1 cervical cancer with tumors ≥ 2 cm? Gynecol Oncol. (2013) 131:87–92. 10.1016/j.ygyno.2013.07.079 23872192

[35] Hazell SZ Stone RL Lin JY Viswanathan AN. Adjuvant therapy after radical trachelectomy for stage I cervical cancer. Gynecol Oncol Rep. (2018) 25:15–8. 10.1016/j.gore.2018.05.001 29977985 PMC6030022

[36] Rob L Skapa P Robova H. Fertility-sparing surgery in patients with cervical cancer. Lancet Oncol. (2011) 12:192–200. 10.1016/S1470-2045(10)70084-X 20619737

[37] Pareja R Rendón GJ Vasquez M Echeverri L Sanz-Lomana CM Ramirez PT. Immediate radical trachelectomy versus neoadjuvant chemotherapy followed by conservative surgery for patients with stage IB1 cervical cancer with tumors 2cm or larger: a literature review and analysis of oncological and obstetrical outcomes. Gynecol Oncol. (2015) 137:574–80. 10.1016/j.ygyno.2015.03.051 25827293

[38] Willows K Lennox G Covens A. Fertility-sparing management in cervical cancer: balancing oncologic outcomes with reproductive success. Gynecol Oncol Res Pract. (2016) 3:9. 10.1186/s40661-016-0030-9 27795832 PMC5073939

[39] Lintner B Saso S Tarnai L Novak Z Palfalvi L Del Priore G . Use of abdominal radical trachelectomy to treat cervical cancer greater than 2 cm in diameter. Int J Gynecol Cancer. (2013) 23:1065–70. 10.1097/IGC.0b013e318295fb41 23722476

[40] Fagotti A Pedone Anchora L Conte C Chiantera V Vizza E Tortorella L . Beyond sentinel node algorithm. toward a more tailored surgery for cervical cancer patients. Cancer Med. (2016) 5:1725–30. 10.1002/cam4.722 27230108 PMC4971900

[41] Wagner AE Pappas L Ghia AJ Gaffney DK. Impact of tumor size on survival in cancer of the cervix and validation of stage IIA1 and IIA2 subdivisions. Gynecol Oncol. (2013) 129:517–21. 10.1016/j.ygyno.2013.03.008 23528928

[42] Pedone Anchora L Bizzarri N Kucukmetin A Turco LC Gallotta V Carbone V . Investigating the possible impact of peritoneal tumor exposure amongst women with early stage cervical cancer treated with minimally invasive approach. Eur J Surg Oncol. (2021) 47:1090–7. 10.1016/j.ejso.2020.09.038 33039294

[43] Casarin J Buda A Bogani G Fanfani F Papadia A Ceccaroni M . Predictors of recurrence following laparoscopic radical hysterectomy for early-stage cervical cancer: a multi-institutional study. Gynecol Oncol. (2020) 159:164–70. 10.1016/j.ygyno.2020.06.508 32665147

