# Supplementary material for: Outcomes of Trachelectomy vs. Hysterectomy for Early-Stage Cervical Cancer: A Systematic Review and Meta-Analysis
Source: Front Surg. 2021 Nov 11;8:735944. doi: 10.3389/fsurg.2021.735944 (PMC8631813; doi:10.3389/fsurg.2021.735944)
Supplement: Supplementary file 2 [file Table_2.DOC]

**Supplementary table 2. Additional details extracted from the included studies**

| **Author (year of publication)** | **Radicality description on parametria** | **Adjuvant treatments provided** | **Lymph nodal surgical assessment** | **Surgical approaches (laparoscopy vs Open) used in cases treated by hysterectomy** |
| --- | --- | --- | --- | --- |
| Beiner M.E et al (2008) | *RT+pelvic lymph node assessment*: resection of a 1–2 cm vaginal cuff; resection of the medial half of the cardinal and uterosacral ligaments; uterine artery preserved; its vaginal branch ligated; cervix transected at the lower uterine segment; mersilene suture placed around the lower uterine segment as a permanent cervical cerclage; vaginal mucosa sutured to the cervical stump (lower uterine segment)  *RH*: ligation of uterine artery at its origin; dissection of ureter from the pubovesical ligament to entry into the bladder; resection of medial ½ of the cardinal and uterosacral ligament and removal of 1-2 cm vaginal cuff; pelvic lymphadenectomy with B/L removal of all fatty lymph node bearing tissue from the lower portion of the common iliac artery to the deep circumflex iliac vein. | Adjuvant radiotherapy provided in 1% of the participants in each of the two groups | In both the groups, no patient had metastases to the pelvic lymph nodes | Laparoscopic (?) |
| Lu Qi et al (2019) | *RT:* Laparoscopic pelvic lymphadenectomy performed; round ligament transected 4 cm from the uterine horns; uterine vessels and ureters identified bilaterally and separated with forceps; ureteral tunnels dissected up to the ureteral orifice and freed from their beds; cul-de-sac of the peritoneum dissected, and rectal vaginal space developed; descending branch of the uterine artery ligated; ascending branch preserved; uterosacral and cardinal ligaments occluded and severed 2 to 3 cm from the cervix and 2 to 3 cm of the vaginal wall exposed; cervix transected approximately 0.5 cm below the level of the isthmus of the uterus, and the vaginal vault transected 2 to 3 cm from the cervix; prophylactic cerclage performed beneath the level of the internal os; reanastomosis of the uterine corpus and upper vagina laparoscopically performed with a continuous running suture; round and uterosacral ligaments and the vesicouterine peritoneum reattached to the neocervix  *RH*: Cervix was grasped with a cervical clamp and a self-made balloon-like solid occluder was placed in vagina; the uterine vessels were coagulated and transected at their origin; the ureters were separated from their median attachments to the peritoneum and then unroofed to the point of their insertion into the bladder; to reduce the risk of port-site metastasis, a tube was put in the vaginal canal, and the specimen removed from the tube to avoid contact with the vaginal wall, and the vaginal were thoroughly irrigated before continuous suturing. | RT: Adjuvant chemotherapy therapy provided in 4.3% participants  RH: 30.1%; radiation (n=9), chemotherapy (n=8), concurrent chemoradiation (n=5) | RT: no lymph node metastasis  RH: lymphatic metastasis in 12.3% subjects | Laparoscopic |
| Marchiole et al (2007) | *RT:* procedure began with a pelvic lymph node dissection followed by making a vaginal cuff, dissecting the bladder and the ureters, opening the Douglas pouch and resecting bilaterally, proximal part of parametria after identification of the uterine artery which was preserved. The cervix was cut 5 mm below the isthmus. The pouch of Douglas was closed and a permanent cerclage was put at the level of the isthmus. The final step was the anastomosis of the vagina and the uterine isthmus.  *RH*: A complete laparoscopic pelvic lymphadenectomy was performed. Laparoscopic dissection was performed in the same way as R, except that the uterine artery was coagulated and cut at its origin. The vaginal steps were the same as in RT until the parametrial dissection. At that time the uterus with the entire uterine arteries was extracted and the original edge was closed with a purse-string suture. | Adjuvant radiotherapy given to 6.7% women in the RT group and to 6.4% women in the RH group. | RT: lymph node status- N0 (95.8%), Micro metastasis (3.4%), macro metastasis (0.8%)  RH: lymph node status- N0 (95.7%), Micro metastasis (2.9%), macro metastasis (1.4%) | Laparoscopic |
| Diaz et al (2008) | *RT*: Delineating an adequate vaginal margin (usually 1–2 cm); circumferentially incised the vaginal mucosa; anterior and posterior vaginal mucosa folded over the ectocervix; inferior portion of uterosacral ligaments divided; develop vesicouterine space and open the paravesical spaces; inferior aspect of the left bladder pillar transected; parametrium is divided between the 2 clamps; cervicovaginal branch of the left uterine artery is doubly clamped, divided, and secured with a suture ligature; subsequently, amputate the cervix; vaginal mucosa re-approximated to the cervical stump | Only one patient in the RT group had required concurrent cisplatin and definitive radiotherapy | RT: lymph node metastasis present in 15%  RH: lymph node metastasis present in 8% | Open |
| Machida et al (2018) | Details of radical trachelectomy and hysterectomy not provided in the manuscript | RT: 3.7% received radiotherapy and 2.6% received chemotherapy  RH: 6.2% received radiotherapy and 3.0% received chemotherapy | Pelvic lymphadenectomy done in 91.1% patients in RT group and in 60.5% in RH group | Not specified |
| Rizzuto et al (2019) | *RT:* Sharp dissection to separate the vagina from adjacent structures; vesicovaginal space was further developed; ureter was mobilised with blunt dissection and retracted cranially; descending vaginal and cervical branches of the uterine artery ligated and divided; uterosacral ligaments were identified, ligated and divided approximately 1-2 cm from the cervix; cardinal ligaments were identified between the Para vesical space anteriorly and the pararectal space posteriorly; 1-2 cm of tissue obtained lateral to the cervix to ensure an adequate margin of resection; cervical cerclage done; vaginal epithelium re-anatomised to the isthmic endocervical epithelium; Bilateral pelvic lymphadenectomy performed. | Not reported | No patients with positive pelvic lymph node; 16% in RT group and 23% in RH group with lympho-vascular space invasion | Open |
| Yoshino et al (2020) | Patients underwent an RT with pelvic lymph node dissection. All procedures were performed by preserving the uterine arteries. Specific details of trachelectomy and hysterectomy not provided in the manuscript. | RT: adjuvant treatment in 21.4% patients; of which in 11.9% there was concurrent chemo-radiotherapy  RH: adjuvant treatment in 36.4% patients; of which in 33.5% there was concurrent chemo-radiotherapy | RT: lymph node metastasis present in 9.5%  RH: lymph node metastasis present in 10.4% | Open |
| Yoshihara et al. (2018) | Details of radical trachelectomy and hysterectomy not provided in the manuscript | Adjuvant chemotherapy in 20% patients in RT and 27.3% patients in RH group | Positive lymph node present in 1 patient each in the two groups; 20% in RT group and 31.8% in RH group with lympho-vascular space invasion | Open |
| Guo et al. (2018) | *RT:* The round ligaments were transected and ligated laterally; paravesicle and pararectal spaces were developed with ligation of uterine arteries and preservation of ovarian vessels; ureter mobilized from the medial sheath of the broad ligament to the level of the cardinal ligament; cervical canal separated from the corpus uteri and opened through a longitudinal section at the 12 o’clock location; frozen section prepared 10 mm from the surgical margin to ensure a 10-mm negative endocervical margin; if frozen sections suggested tumor-free surgical margins, uterus was reconstructed to the upper vagina with absorbable sutures; after the incision was taken down and the specimen was removed, a cerclage suture performed | Adjuvant chemotherapy in 37.1% patients in RT and 36% patients in RH group | 18.2% in RT group and 18.8% in RH group with lympho-vascular space invasion | Open |
| Van Gent et al. (2014) | *RT*: Complete pelvic lymphadenectomy (removal of common and external iliac nodes and lymphatic tissue in the obturator loge above the obturator nerve); dissection of the bladder from the cervix and vagina; opening of the peritoneum of the pouch of Douglas and blunt and sharp development of the prerectal space; dissection of the hypogastric nerves from the medial leaf of the peritoneum posterior the ureter; dissection of the ureter from the medial leaf of the peritoneum; clamping and cutting the peritoneal flap medially attached to the rectum at the level of halfway the circumference of the rectum; clamping and cutting of the uterosacral ligaments lateralizing the ureter and the hypogastric plexus; separation of the mesometrium and the mesenterium of the bladder; dissection of the uterine artery from the parametrium until its diversion in an ascending and descending branch close to the uterus at the level of the isthmus; dissection of the ureter through its passage in the ureter channel until its entrance in the bladder; dissection and resection of the mesometrium from the internal iliac artery medially using the deep uterine vein as posterior border, saving the uterine artery and its ascending branch; cleavage of the uterus at the level of the isthmus just distally of the entry of the ascending branch of the uterine artery; Dissection of the paracolpium until 2-cm vaginal margin obtained; clamping of the vaginal vault, cutting the vagina below these clamps; cervix with vaginal margin, mesometrium wings, and long-tailed sacrouterine ligaments removed; cerclage in the neocervix, placement of uterine catheter; attachment of the neocervix to the vagina using interrupted absorbable stitches  *RH:* Transect the round ligaments; open the peritoneum of the broad ligament above the level of the common iliac arteries; dissect the ureters from as cranially as possible up to the entrance of the ureteral canal; lymphadenectomy along the external iliac vessels, the common iliac artery, the hypogastric artery, from the obturator fossa and the presacral area; area lateral to and underneath the superior vesical arteries (“lateral parametrium”) is cleared from the lymphatic tissue; separate the uterine vessels from the underlying bladder mesentery; transect the uterine vessels at their origin; develop the presacral space; identify the hypogastric nerve system attached to the subperitoneal and dense connective tissue and dissect it laterally; open the bladder peritoneum, cut the supravaginal septum and dissect the bladder down from the cervix and the vagina; remove the ureter from the posterior leaf of the vesico-uterine ligament; place clamps on the vagina and cut the vagina below these clamps; remove the uterus with the mesometrium wings and the long-tailed uterosacral ligaments; close the vaginal cuff with interrupted resorbable sutures | None of the women in both the groups needed adjuvant treatment after surgery | 36% in RT group and 25% in RH group with lympho-vascular space invasion; mean number of nodes removed were 20.6 and 23.5 in RT and RH groups respectively | Unclear |
| Zhang et al. (2014) | *RT:* The round ligaments were transected and ligated laterally; paravesicle and pararectal spaces were developed with ligation of uterine arteries and preservation of ovarian vessels; ureter mobilized from the medial sheath of the broad ligament to the level of the cardinal ligament; cervical canal separated from the corpus uteri and opened through a longitudinal section at the 12 o’clock location; frozen section prepared 10 mm from the surgical margin to ensure a 10-mm negative endocervical margin; if frozen sections suggested tumor-free surgical margins, uterus was reconstructed to the upper vagina with absorbable sutures; after the incision was taken down and the specimen was removed, a cerclage suture performed | RH patients (52.2%) were more likely to receive postoperative chemotherapy or radiation compared with RT patients (36.11%) | The parametrial lymph nodes found in 77.8% patients in the RT group and in 95.6% patients in the RH group. Solitary LN metastases observed in 10.71% patients in the RT group and in 6.9% patients in the RH group. | Open (?) |
| Li X et al. (2016) | *RT:* Full pelvic lymphadenectomy performed; round ligaments were transected and ligated laterally; paravesicle and pararectal spaces were developed; ureter was mobilized from the medial sheath of the broad ligament to the level of the cardinal ligament; uterine artery was identified at its origin from the internal iliac artery; ureter was identified just inferior to the uterine artery; vaginal branch of the uterine artery was ligated; transection of the cervix at the level of the internal cervical os or a circumferentially performed colpotomy; for most of the patients no attempt to spare the internal cervical os was made. | Adjuvant treatment in 29% patients in RT and 34% patients in RH group | 17.8% in RT group and 19.1% in RH group with lympho-vascular space invasion | Not specified |
